# Polyphenolic Characterization of Merlot, Tannat and Syrah Skin Extracts at Different Degrees of Maturity and Anti-Inflammatory Potential in RAW 264.7 Cells

**DOI:** 10.3390/foods10030541

**Published:** 2021-03-05

**Authors:** Nawel Benbouguerra, Josep Valls-Fonayet, Stephanie Krisa, François Garcia, Cédric Saucier, Tristan Richard, Ruth Hornedo-Ortega

**Affiliations:** 1 SPO, Université de Montpellier, INRAe, Montpellier Supagro, 34000 Montpellier, France; nawal.benbouguerra@gmail.com (N.B.); francois.garcia@umontpellier.fr (F.G.); cedric.saucier@umontpellier.fr (C.S.); 2 Axe Molécules d’Intérêt Biologique, Unité de Recherche Œnologie, ISVV, EA 4577, USC 1366 INRA Université de Bordeaux, 210 Chemin de Leysotte, 33882 Villenave d’Ornon, France; josep.valls-fonayet@u-bordeaux.fr (J.V.-F.); stephanie.krisa@u-bordeaux.fr (S.K.); tristan.richard@u-bordeaux.fr (T.R.)

**Keywords:** phenolic compounds, *Vitis vinifera*, ripening, grapes, anti-inflammatory, antioxidant activity

## Abstract

(1) Background: Both sensory quality and healthy attributes of *Vitis vinifera* grapes used for winemaking are closely related with the polyphenolic composition of their skins. (2) Methods: In this study, the polyphenolic characterization (flavan-3-ols, procyanidins, flavonols, stilbenes, anthocyanins) was investigated by ultra performance liquid chromatography coupled to a triple quadrupole mass spectrometer (UPLC-QqQ-MS). Skins from *Vitis vinifera* Merlot, Tannat, and Syrah red grape varieties cultivated in the south of France at different stages of ripening in 2018 were used. The anti-inflammatory and the antioxidant potential of the extracts were evaluated by the measure of nitric oxide (NO) and the intracellular reactive oxygen species production (ROS) in lipopolysaccharide (LPS)-stimulated macrophages. (3) Results: 41 polyphenols were quantified in all samples. Generally, the flavan-3-ol and procyanidin content decreased during ripening whereas the anthocyanins and stilbenes increased. In addition, as a novelty of this work, a wide identification and characterization of monomeric and oligomeric stilbenes was assessed by using authentic standards isolated in our laboratory, some of them (parthenocissin A and miyabenol C) reported for the first time in Merlot, Tannat and Syrah cultivars. The before-veraison skin extracts of all studied varieties, exhibited higher NO and ROS productions inhibition (>50%) proving both antioxidant and anti-inflammatory properties.

## 1. Introduction

The cultivation of the vine is one of the largest crops in the world. Recent data reported in 2018, estimates that the area under cultivation for the production of wine grapes, table grapes or dried grapes is 7.4 million hectares. Specifically, the production of wine from *Vitis vinifera* grapes reached 292 million hectoliters in 2018. *V. vinifera* species are characteristic for their great diversity. In fact, the actual number of *V. vinifera* varieties is estimated at 6000 [1].

Polyphenolic compounds are secondary metabolites with undeniable demonstrated biological properties [2,3]. These compounds are usually divided in two important families: Flavonoids (flavan-3-ols, procyanidins, flavonols, anthocyanins) and non-flavonoids (phenolic acids and stilbenes) [4]. Grapes are especially rich in these compounds, and the polyphenolic content and composition varies substantially depending on grape variety, stage of ripening, temperature, soil, fungal infection or radiation and soil salinity [5,6]. Moreover, the polyphenolic composition is closely related to the sensory properties of wine such as color, mouthfeel, astringency, and bitterness) [7]. In general, red grape skins are initially rich in flavan-3-ols and procyanidins at pre-veraison state whereas at veraison and maturity stages anthocyanins are the major compounds. The greater quantities of stilbenes were found at maturity stage [8,9,10]. 

The moderate consumption of red wine, characteristic of the Mediterranean diet, and their richness in polyphenols has been broadly associated to the health benefits of this dietary pattern [11,12]. Consequently, the study of polyphenolics from grapes and red wines have enthralled the attention of scientists to define their chemical composition and properties concerning human health. Indeed, grape polyphenols have evidenced beneficial effects to prevent neurodegenerative, cardiovascular and metabolic disorders and certain types of cancers [13,14]. Oxidative stress and inflammation are important pathological hallmarks related with the development and progression of several diseases [15]. Therefore, in order to counteract this reaction, there is a growing interest to identify grape anti-inflammatory and antioxidant compounds. 

In this study, the polyphenolic characterization of flavan-3-ols, procyanidins, flavonols, anthocyanins and stilbenes by Ultra Performance Liquid Chromatography coupled to a triple quadrupole mass spectrometer (UPLC-QqQ-MS) and UPLC-photodiode array (PDA) of skins from *V. vinifera* Merlot, Tannat, and Syrah red grape varieties cultivated in the south of France at different stages of ripening on 2018 (before veraison, veraison and maturity) were evaluated. Thanks to the available monomeric and oligomeric authentic stilbenes standards, isolated and purified in our laboratory, a wide and accurate characterization of these compounds was assessed. In addition, the anti-inflammatory and the antioxidant potential of extracts on LPS-stimulated macrophages (RAW 264.7 cells) were determined. 

## 2. Materials and Methods 

### 2.1. Chemicals and Reagents

Folin Ciocalteu phenol reagent, sodium carbonate, 1,1-diphenyl-2-picrylhydrazyl (DPPH), Trolox, phloroglucinol, ascorbic acid, tartaric acid, sodium hydroxide, hydrochloric acid, lipopolysaccharide (LPS), Roswell Park Memorial Institute medium (RPMI) and Dulbecco’s Modified Eagle Medium (DMEM) mediums, fetal bovine serum (FBS), Griess reagent, 2′7′-dichlorodihydrofluoroscein diacetate acetyl (DCFH_2_-DA), 3-(4,5-dimethylethiazol-2-yl)-2,5-diphenyl tetrazolium bromide (MTT), dimethyl sulfoxide (DMSO), glutamine, gallic acid, catechin, malvidin-3-*O*-glucoside, *trans*-piceid and *trans*-resveratrol were obtained from Sigma Aldrich (Steinheim, Germany). *Trans*-astringin was purchased from Carbosynth (Berkshire, UK) and *trans*-piceatannol from ChromaDex (Los Angeles, CA, USA). Acetonitrile, methanol and water LC-MS were obtained from Biosolve (Dieuze, France) and trifluoroacetic acid and sodium acetate were purchased from Carlo Erba (Peypin, France). RAW 264.7 cells were provided by ATCC (Manassas, VA, USA). ε-viniferin, δ-viniferin, ω-viniferin, pallidol, parthenocissin A, miyabenol C, hopeaphenol and isohopeaphenol were isolated from a grapevine raw shoot in our laboratory. The *cis*-isomers stilbenes were obtained from *trans*-isomers using by applying Ultraviolet-C irradiation (254 nm).

### 2.2. Grape Samples

Merlot, Tannat and Syrah red *V. vinifera* varieties were cultivated and harvested on the INRA*e* Montpellier vineyard (43°37′02.7″ N 3°51′22.3″ E, average annual temperature: 16.38 °C, average annual precipitation: 1063.5 mm and soil: Gravels and river sand) at different ripening stages: Before veraison (18 June 2018), veraison (27 July 2018) and maturity (3 September 2018). Grapes clusters were frozen within an hour after sampling at −80 °C until sample preparation.

### 2.3. Grape Samples Preparation and Polyphenolic Extraction

Grapes were thawed and pH and °Brix were measured. For this, 50 berries were crushed, the pH was determined by a multi-parameter analyzer (Consort C3010) and the concentration of sugar was determined using a hand-held refractometer (OPL-FOCA) (results expressed as °Brix).

Skins of 30 grapes of each variety (Merlot, Tannat and Syrah) were manually separated from the pulp. In order to extract the polyphenolic compounds a solid/liquid extraction with acetone/water (70:30; *v*/*v*) were performed. After mixing during 18 h in the dark, the sample was filtered (0.45 µm filter paper) and concentrated in a rotavapor under low pressure fixed at 37 °C. Finally, the obtained extract was freeze-dried and stored at −80 °C until further analysis.

### 2.4. Determination of Total Polyphenol Content (TPC)

The total phenolic content of samples was determined by the Folin–Ciocalteu method [16]. A stock solution of 5 g/L was prepared by diluting 5 mg of skins extracts in 1 mL of methanol. Twenty microliters of the diluted extract was mixed to 100 μL of diluted Folin-Ciocalteu reagent and 80 μL of sodium carbonate solution (7.5%). The prepared samples were thoroughly shaken and let sit for 30 min at room temperature in the darkness. Finally, the absorbance was measured at 760 nm using a microplate reader (FLUOstar Optima, BMG Labtech). A calibration curve of gallic acid (GAE) (0–0.5 mg/mL) was used to determine the concentration of polyphenols in samples. The results were expressed as g of gallic acid equivalents (GAE) per g of skin grapes fresh weight (FW).

### 2.5. Determination of Radical Scavenging Activity (DPPH˙ Assay)

The DPPH assay was used to determine the antioxidant activity of the extracts [17]. To this end, a stock solution was prepared by diluting 5 mg of skins extracts in 1 mL of methanol. Fifty microliters of this stock solution was diluted in methanol (4 times) and mixed with 150 μL of a DPPH (200 μM in methanolic solution) and then incubated during 20 min in the dark at room temperature. Finally, the absorbance was measured using a microplate reader set at 517 nm (FLUOstar Optima, BMG Labtech). A calibration curve of Trolox (TE) (0–300 µM). The results were expressed as µM TE/g of skin grapes FW.

### 2.6. Analysis of Proanthocyanidins Following Acid Catalysis with Phloroglucinol

The analysis of proanthocyanidins were carried out by UPLC-PDA (Waters Acquity). The proanthocyanidin assay was carried out based on the method described by Kennedy et al. [18]. A solution of 0.1 N of HCl in methanol, containing 50 g/L phloroglucinol and 10 g/L ascorbic acid was prepared. Samples (at 10 g/L in methanol) were combined with this solution during 20 min at 50 °C, and then mixed with 40 mM aqueous sodium acetate solution (5 volumes) to stop the reaction. Samples were finally filtered with polytetrafluoroethylene (PTFE) 0.45 μm filters and injected. An Acquity UPLC ethylene bridged hybrid (BEH) C18 column (2.1 mm × 50 mm, 1.7 μm particle size) thermostated at 40 °C was used to analyze phloroglucinol adducts. The flow rate was at 0.45 mL/minute and the injection volume was 7.5 µL. Solvent A consisting in water/TFA (99:1 *v*/*v*) and solvent B, acetonitrile (100%) were used as mobile phases. The programmed gradient was: 0 min 2% B, 8 min 6% B, 14 min 20% B, 16 min 99% B, and 20 min 2% B. In order to calculate the mean Degree of Polymerization (mDP), the sum of all subunits (flavan-3-ol monomers and phloroglucinol adducts expressed in millimoles) was divided by the sum of all flavan-3-ols monomers (expressed in millimoles).

### 2.7. Individual Determination of Phenolic Composition

#### 2.7.1. Momonomeric Flavan-3-ols, Procyanidins, Flavonols and Stilbenes

The momonomeric flavan-3-ols, procyanidins, flavonols and stilbenes analyses were performed by UPLC-QqQ-MS based on a previous method [19]. Both skin samples and pure compounds were solubilized in methanol/water (1:1; *v*/*v*) at 20 g/L concentration. The instrument used for the analysis was a UPLC (Agilent Technology 1260 Infinity, Agilent Technologies, Santa Clara, CA, USA), coupled to a Triple Quadrupole Detector (Agilent Technologies 6430). A Poroshell 120 EC-C18 column (150 mm × 2.1 mm, 2.7 µm particle size) was used as a stationary phase. The method used a binary gradient, A (water/0.1% formic acid) and B (acetonitrile/0.1% formic acid), programmed in the following gradients: 5–17.5% B (0–5 min), 17.5–33% B (5–7.5 min), 33% B (7.5–10 min), 33–40% B (10–15 min), 40–95% B (15–16 min), 95% B (16–19 min) and 5% B (19–21 min). The flow rate and the column temperature were fixed at 0.3 mL/minute and 35 °C, respectively. The injection volume was 4 µL. Multiple reaction monitoring (MRM) mode with specific transitions for each polyphenolic compound was used to detection purposes. A calibration curve ranging from 0.05 to 26 mg/L was prepared in methanol/water (1:1; *v*/*v*) with pure standards. Samples were analyzed in triplicate and the results were expressed as mg per kg of skin grapes FW.

#### 2.7.2. Anthocyanins

The anthocyanins analyses were carried out with the same column and solvent used for proanthocyanidin analyses (see Section 2.6). Skin extracts were solubilized at 10 g/L in MeOH/eau (80:20; *v*/*v*), filtrated through PTFE 0.45 μm filters then injected. The flow rate was fixed at 0.25 mL/minute and the temperature set at 50 °C. The mobile phases consisted of solvent A (water/TFA 99:1 *v*/*v*) and solvent B (acetonitrile 100%) scheduled in the following gradient: 0 min 1% B, 5 min 8.8% B, 30 min 20.6% B, 34 min 96% B, 34.1 min 1% and 40 min 1% B. Eluting peaks were monitored at 520 nm. A calibration curve of malvidin-3-*O*-glucoside (0–200 mg/L) was used to quantify anthocyanins in all samples (all anthocyanins are expressed as malvidin-3-*O*-glucoside) [20].

### 2.8. Cell Culture and Treatment

RAW 264.7 cells were cultured in DMEM containing 10% of FBS and maintained at 37 °C with 5% of CO_2_ in a humidified incubator. Cells were subcultured at a density of 50,000 cells per well, in 96-well culture plates with 200 µL of culture medium. After 24 h, cells were incubated with skin extracts (50–300 µg/mL) in RPMI medium supplemented with glutamine (4 mM) in presence or absence of LPS (0.1 µg/mL) (200 µL final volume per well).

### 2.9. MTT Cell Viability

MTT test, a quantitative and consistent colorimetric assay was carried out to assess the cell viability of cells [21] after being treated with skin extracts. After 24 h of treatment, RAW 264.7 cells were incubated with 0.5 mg/mL of MTT during 3 h at 37 °C. The crystals formed at the well bottom were dissolved with 100 µL of DMSO. After 30 min of incubation in darkness, the absorbance was measured at 595 nm by using a microplate reader (FLUOstar Optima, BMG Labtech).

### 2.10. Intracellular NO Measurement

In the same way and after 24 h of treatment, 50 µL of supernatant mixed with 50 µL of Griess solution. After 15 min in darkness, the absorbance was measured at 550 nm using a microplate reader (FLUOstar Optima, BMG Labtech). A calibration curve of NO_2_ (0–100 µM) was used. Data were expressed as NO production (µM) compared with cells treated only with LPS (positive control).

### 2.11. Intracellular Reactive Oxygen Species (ROS) Measurement

Generation of intracellular ROS in cells was analyzed using a fluorometric probe: DCFH_2_-DA. After treatment, cells were washed with PBS and then 150 µL of DCFH_2_-DA (10 µM) was added. After 30 min at 37 °C, the fluorescence intensity was quantified using a microplate reader (FLUOstar Optima, BMG Labtech) with a wavelength of excitation and emission of 485 nm and 520 nm respectively. All experiments were performed in darkness. Results were given as ROS production (fluorescence intensity) compared with cells treated only with LPS (positive control).

### 2.12. Statistical Analysis

The data were subjected to one-way ANOVA test with XLSTAT version 19.02. Comparison between the different stages of ripening was performed using Tukey’s test and *p* < 0.05 was considered significant.

## 3. Results and Discussion

Grapes of Merlot, Tannat and Syrah cultivars were harvested at different stages of ripening: June 2018 (Before Veraison; BV), July 2018 (Veraison; V) and September 2018 (Maturity; M). Table 1 summarizes the °Brix and pH values of samples.

### 3.1. TPC and Antioxidant Activity of Skins of Merlot, Tannat and Syrah Cultivars at Different Stages of Ripening

Table 2 summarizes the TPC expressed as g GAE per kg FW from the skins of Merlot, Tannat and Syrah grapes at the three different ripening stages (BV; V and M). The highest TPC was observed at BV stage in all cultivars (18.29–19.16 g GAE/kg FW). These values decreased at V and M stages for all cultivars. These results are in agreement with previous works that observed that TPC decreases when the berry weight increases [22,23]. The same tendency was observed for antioxidant activity calculated by DPPH test (expressed as g TE/kg of skins FW) (Table 2). In fact, a decrease of 28–56% on the antioxidant activity was noticed between BV and M. In conclusion, TPC is in accordance with DPPH, the higher polyphenol concentration corresponded to the higher antioxidant activity (Table 2).

### 3.2. Individual Polyphenolic Characterization of Skins of Merlot, Tannat and Syrah Cultivars at Different Stages of Ripening

**Figure 1:**
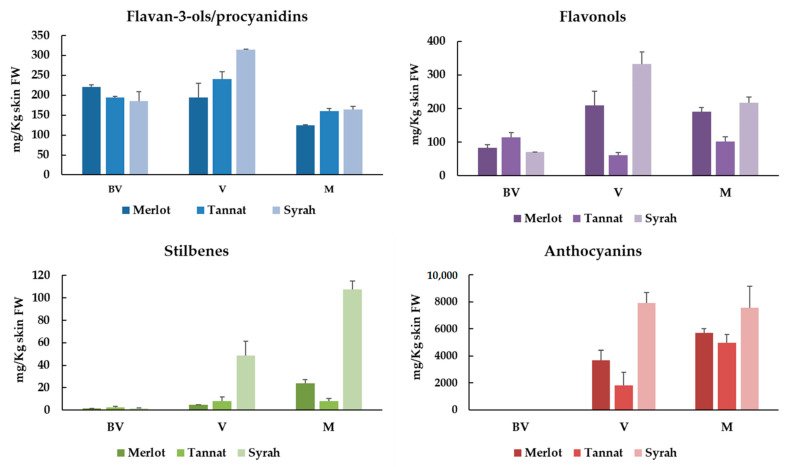
Sum of the individual flavan-3-ols/procyanidins, flavanols, stilbenes and anthocyanins of Merlot, Tannat and Syrah skin extracts at different ripening stages (BV: Before veraison; V: Veraison; M: Maturity).

Figure 1 displays the sum of the individual polyphenolic compounds (flavan-3-ols and procyanidins, flavonols, stilbenes and anthocyanins) expressed as mg/kg of skins FW of Merlot, Tannat and Syrah extracts at the three ripening stages (BV, V and M). In addition, Table 3 and Table 4 summarize the individual polyphenols (Figures S1 and S2) identified and quantified in all samples. A total of 41 compounds have been identified: 6 flavan-3-ols and procyanidins; 7 flavonols; 16 stilbenes and 12 anthocyanins.

#### 3.2.1. Monomeric Flavan-3-ols and Procyanidins

Monomeric flavan-3-ols and procyanidin are especially relevant in grapes and wine for their contribution on color stabilization and their astringent and bitter properties [24]. They are especially located in all grape clusters’ solid parts: Skins, seeds and stalks and their amounts vary during ripening, normally reaching their maximal levels around veraison [25]. In our samples, the highest levels were observed at V stage (195–315 mg/kg skin FW). These values diminished significantly at M stage (between 33–48%) (Figure 1).

Regarding flavan-3-ols monomers individually, (+)-catechin is the major compound followed by (−)-epicatechin at BV stage. (+)-catechin quantities diminished considerably during ripening in all varieties (Table 3). This tendency has been observed in previously studies for the variety Syrah, [26] Tannat [22] and Merlot [23]. Interestingly, (−)-epicatechin levels increased from BV to V stage to finally diminished at M stage. In any case, the final amounts of (−)-epicatechin are significantly higher that the initials (BV stage). This observation was also in accordance with other studies [22,23].

Four B-type procyanidins were identified and quantified in all samples: B1, B2, B3 and B4. Among them, procyanidin B1 (epicatechin-(4β→8)-catechin) was the predominant with initial values (BV) ranging from 50.77–75.53 mg/kg skins FW. The major levels for this compound were observed at the V stage to finally decrease at the M stage. Important amounts of procyanidin B3 were also present in all samples, which also decreased over the course of maturation in all cultivars and in all ripening stages. Finally, procyanidins B2 and B4 were also present in all samples with levels between 4.5 and 9.57 mg/kg of FW (Table 3). In accordance with our study, the procyanidin B1 was quantified as the major compound in Syrah skin extracts at maturity [26]. In a previous study, these four B-type proanthocyanidins have also been quantified in Merlot skins extracts at different maturity stages, however procyanidin B2 was the predominant [23].

In order to get more information about the oligomeric and polymeric proanthocyanidins (also named condensed tannins) the mean degree of polymerization (mDP) of skin extracts was calculated by means of phloroglucinolysis. It can be noted that mDP values had a tendency of increasing over ripening for Tannat and Syrah skin extracts (Table 4). At M stage, Syrah showed the highest mDP values very closely followed by Merlot and Tannat. The same trend has been previously described in Carménère, Merlot, Cabernet Franc and Cabernet Sauvignon skin extracts at different ripening stages [10]. In addition, it is also worth noting that mDP values in grape skin extracts can vary substantially. For example, for Cabernet Sauvignon grapes, skin tannin mDP can range from 3.4 to 85.7 [27].

#### 3.2.2. Flavonols

Flavonols are important polyphenolic compounds that vary in color from white to yellow and play a crucial role in the color stabilization (through copigmentation with anthocyanins) in young red wines. These compounds are also implicated in the perception of in-mouth astringency and bitterness [28,29]. Usually, flavonols are located in berry skins of both white and red grapes, and as other grape polyphenols, their content varies considerably depending on cultivars and state of ripeness [25]. The same tendency observed for total flavan-3-ols was noticed for flavonols. For Syrah and Merlot cultivars, the greater values were noted at V stage (209–332 mg/kg) whereas for Tannat they are observed at BV stage (114 mg/kg) (Figure 1).

As can be expected, the individual flavonols identified in skin samples were found in 3-*O*-glycoside forms. Among them, quercetin-3-*O*-glucuronide was the most prevalent flavonol in all varieties. However, different tendencies can be observed between varieties. Indeed, in Merlot and Syrah cultivars their amounts increased greatly at V stage then diminished at M stage (although the M stage quantities remain more elevated that at BV). On the contrary, the major levels of quercetin-3-*O*-glucuronide were observed at BV stage for Tannat skin extracts (Table 3). Substantial amounts of quercetin-3-*O*-glucuronide have also been found in skins extracts of Merlot, Cabernet Sauvignon, Petit Verdot, Syrah, Tempranillo, Garnacha and Garnacha Tintorera [30]. Quercetin aglycone and other quercetin glycosides (3-*O*-hexoside, 3-*O*-galactoside, 3-*O*-rhamnoside and 3-*O*-rutinoside) are also present in small quantities in all samples (Table 3). In addition to that, kaempferol-3-*O*-glucoside has also been quantified and their amounts increase during ripening but only for Syrah variety.

#### 3.2.3. Stilbenes

Stilbenes, polyphenolic compounds that belong to the non-flavonoid family, are present in grapes at very low concentrations (1%) and are mainly concentrated in skins [31]. However, due to their importance as bioactive compounds for plant and human health, their characterization on grapes and other foodstuffs is especially interesting.

The stilbene content varies significantly versus the ripening stages and the maximal concentrations of stilbenes in grape skin are reached at maturity stage (Figure 1). This augmentation is closely related with the expression increase of key enzymes responsible for stilbene synthesis and accumulation: Stilbene synthase, phenylalanine ammonia-lyase and 4-coumarate-CoA ligase [32]. In accordance with this, our results indicated that the total stilbenes increase at V stage to reach their maximum at M for all studied varieties. Between cultivars, Syrah was the variety richer in stilbenes with 107.63 mg/kg skins followed by Merlot (24.11 mg/kg skins) and Tannat (8.17 mg/kg skins) (Figure 1).

A great variety of stilbenes were identified in all samples (Table 3). The authentic standards of several stilbenes (ε-viniferin, δ-viniferin, ω-viniferin, partenocissin A, miyabenol C, hopeaphenol and isohopeaphenol), isolated and purified in our laboratory were used for identification and quantification purposes. Among them, four monomers (resveratrol, piceid, piceatannol and astringin (in their -*trans* and –*cis* configurations)), five dimers (ε-viniferin, ω-viniferin, σ-viniferin, pallidol and partenocissin A), one trimer (miyabenol C) and two tetramers (hopeaphenol and isohopeaphenol) were identified. Piceid (mainly in the –*cis* form) represents the major compound in all varieties (ranging from 0.03–49.64 mg/kg skins) followed by astringin, piceatannol and resveratrol. Important quantities of ε-viniferin were also quantified in samples (0.12–4.77 mg/kg skins). Concerning other oligomeric stilbenes, several differences were observed between cultivars. In Merlot, parthenocissin A was the main oligomeric stilbene but for Syrah it was pallidol. Very similar results have been recently published in Tannat variety at different ripening stages during 2017 harvest [33]. As far as we know, this is the first time that an identification and quantification of complex stilbenes with authentical standards was carried out in Merlot and Syrah varieties. In addition, partenocissin A and miyabenol C were identified for the first time in Merlot, Tannat and Syrah cultivars.

#### 3.2.4. Anthocyanins

Anthocyanins, polyphenolic compounds responsible for the red color of wine, are mainly accumulated in skins and their amounts grows with the fruit maturation degree. These compounds are presents in red grapes during ripening but also in the flesh of “teinturier” varieties [34].

In red grape skins, anthocyanins were the more predominant polyphenolic compounds. In fact, they represented between 84–91% at V and between 94–95% at M in Merlot, Tannat and Syrah varieties (Figure 1). Once again, Syrah skin extract was the richest in anthocyanins with values of 7578 mg/kg skins at maturity, followed by Merlot (5707 mg/kg skins) and Tannat (4972 mg/kg skins) (Figure 1). Fifteen individual anthocyanins, divided in three families (five 3-*O*-glucosides, five 3-*O*-(6-*O*-acetyl)-glucosides and five 3-*O*-(6-*O*-*p*-coumaroyl)-glucosides) were identified in all samples (Table 5) (Figure S2). It is well established in *V. vinifera* red skin grapes that the 3-*O*-glucosides are the main forms followed by 3-*O*-(6-*O*-acetyl)-glucosides and 3-*O*-(6-*O*-*p*-coumaroyl)-glucosides). Delphinidin, cyanidin, petunidin, peonidin and malvidin-3-*O*-glucosides were quantified. Additionally, petunidin, peonidin and malvidin-3-*O*-(6-*O*-acetyl)-glucosides and delphinidin, cyanidin, peonidin and malvidin -3-*O*-(6-*O*-*p*-coumaroyl)-glucosides were also quantified in all samples. Malvidin-3-*O*-glucoside was the major anthocyanin followed by peonidin-3-*O*-glucoside in Merlot skin extracts. However, in the case of Tannat and Syrah, petunidin-3-*O*-glucoside was the second-most predominant anthocyanin. These results were in accordance with other authors that have noticed the same observation in Merlot [10], Tannat [22] and Syrah [26].

### 3.3. Anti-Inflammatory and Antioxidant Potential In Vitro Effects of Skins Extracts of Merlot, Tannat and Syrah Cultivars at Different Stages of Ripening

The anti-inflammatory potential of skins extracts of Merlot, Tannat and Syrah at BV, V and M was assessed by the intracellular measure of nitric oxide (NO), and ROS in a macrophage model cell line (RAW 264.7). NO levels are a well-established marker for the inflammatory process. Indeed, after infection, macrophages are stimulated and can secrete NO and several interleukins in order to destroy the infectious agent. While inflammation is *a priori* a beneficial response, when an excessive production of ROS is chronically generated, the consequence is an important damage at cellular level. In fact, NO is one of the major contributors to the formation of ROS [35]. Thus, chronic inflammation and oxidative stress are considered as a major cause of age-related diseases and cancer [15].

**Figure 2:**
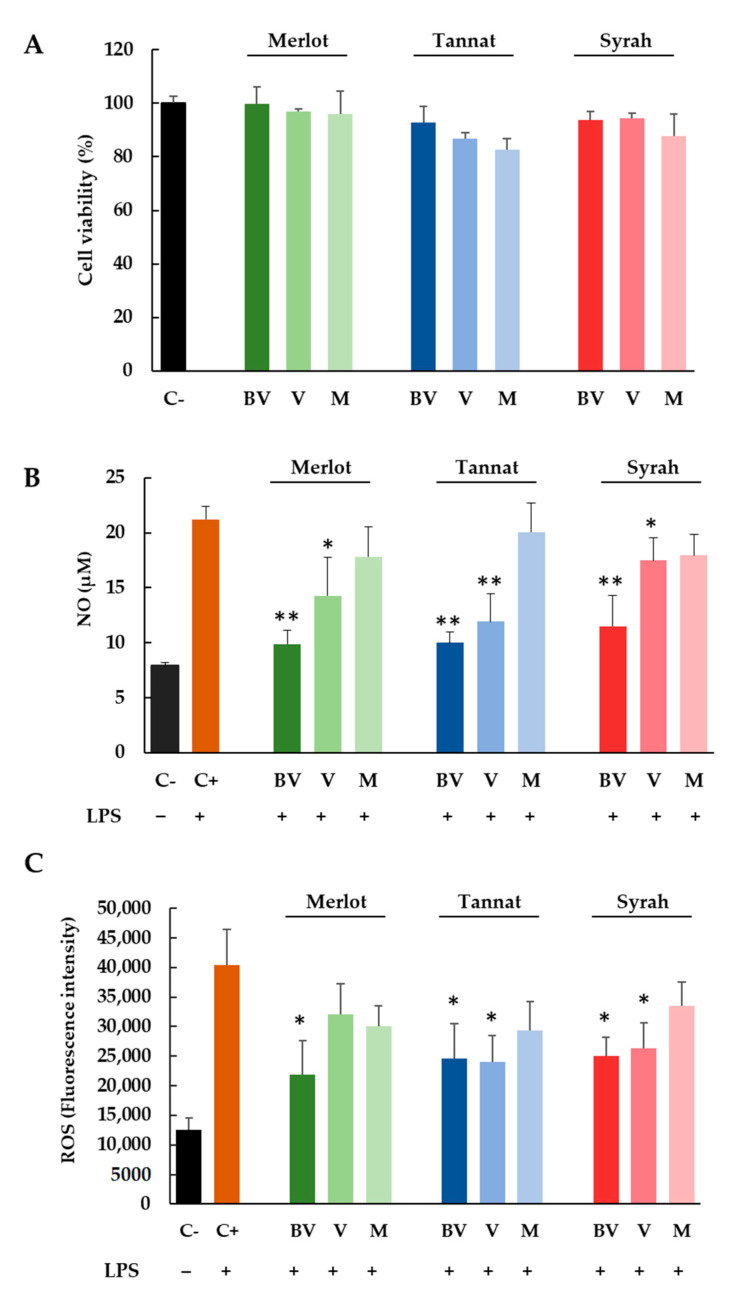
Cell viability (%) (**A**), NO (µM) (**B**) and ROS (fluorescence intensity) (**C**) production in RAW 264.7 cells. Cells were treated for 24 h by LPS (0.1 μg/mL; + control) or LPS with Merlot, Tannat, and Syrah extracts (100 μg/mL) at BV, V and M stages. Results are expressed as mean SEM of four replicates (*n* = 4). * *p*: 0.01–0.05 extracts *versus* + control, ** *p*: 0.001–0.01 extract *versus* + control.

Initially, the cytotoxicity of skin extracts was determined by using the MTT assay, a test based on the reduction of a yellow tetrazolium salt (MTT) to purple formazan crystals by metabolically active cells. For this, cells were treated with different concentrations of skin extracts (50–300 μg/mL) at all experimental conditions (three varieties and three ripening stages) during 24 h. After analyzing the results, the concentration of 100 μg/mL was selected for further analysis to be non-toxic for the cellular model (Figure 2A).

For NO measurement, RAW 264.7 cells were activated with LPS (0.1 µg/mL) in the presence of 100 μg/mL of all skins extracts (three cultivars and three ripening stages). After 24 h of exposure, culture media were analyzed for nitrite (NO_2_) content by the Griess reaction. Figure 2B shows the NO production (µM) for cells without treatment and for cells treated with LPS alone or with LPS and skin extracts. As can be expected, the NO concentration for positive control (cells treated with LPS) increased threefold (21.2 µM) in comparison with the negative control (7.9 µM) (cells without LPS treatment). When cells were also treated with BV and V skin extracts, the NO concentration diminished significantly. In all cases, the most anti-inflammatory sample was the BV extract for all varieties, with NO concentrations below 12 µM, close to the value of the negative control.

In addition, in order to gain insight into the antioxidant potential of skin extracts, the ROS production induced by LPS was investigated by employing the fluorometric probe DCFH_2_-DA that is a widely used indicator to detect and quantify the intracellular produced ROS. Figure 2C displays the effect exerted by skin extracts after 24 h of treatment on ROS production (expressed as fluorescence intensity). As can be clearly observed, the treatment of cells with LPS corresponds to a marked increase of fluorescence (about four times higher) in comparison with negative control proving that the oxidant effect of LPS. However, and in the same way as that for NO production, the treatment with LPS + skin extracts produced a significant reduction of ROS production for BV samples of Merlot and for BV and V samples of Tannat and Syrah varieties (Figure 2C).

Although the individual characterization shows that in general flavan-3-ols, procyanidins, flavonols and stilbenes reached the highest values at V and M stage, it should be taken in consideration that other polyphenolic compounds not identified and quantified in our samples can be the responsible of the observed effects. In fact, TPC values are much higher than the sum of all quantified polyphenols, a fact that can explain that BV samples are generally the most anti-inflammatory and antioxidant extracts. Thus, other bioactive molecules not identified in this work can also contribute to the effect.

Among polyphenols, (+)-catechin which is one of the main skin flavan-3-ol at BV stage has shown similar effects in LPS-stimulated RAW 264.7 cells. Thus, (+)-catechin has demonstrated to be able to suppress the NO release by two different pathways, through direct NO scavenging activity and by inhibiting the nitric oxyde synthase (iNOS) protein expression [36]. In addition, other flavan-3-ol monomers and procyanidins have displayed similar effect in interferon-γ-stimulated macrophages [37]. Taking into account that the major polyphenols in our extracts at V and M stages are the anthocyanins, we can hypothesize that they can be also the responsible molecules of the observed anti-inflammatory and antioxidant effects. This theory can be based on previously results that have demonstrated that malvidin-3-*O*-glucoside (the major anthocyanin in grape skins) was able to attenuate LPS-induced nuclear factor-KappaB (pro-inflammatory transcription factor associated with NO and interleukin liberation) and ROS production in macrophages [38].

## 4. Conclusions

In summary, this work provides a wide characterization of individual polyphenolic compounds (41 compounds) in grape skin extracts. In this work, and thanks to the available oligomeric authentic stilbenes standards, isolated and purified in our laboratory, a wide and accuracy identification and quantification of these compounds was assessed in Merlot and Syrah varieties. Moreover, parthenocissin A and miyabenol C have been identified in Merlot, Tannat and Syrah cultivars for the first time. Although the major polyphenols content was displayed at BV stage, the HPLC-MS analysis has allowed us to monitor the individual evolution and changes in these compounds during ripening. Generally, anthocyanin, flavonol and stilbene contents increased during ripening. However, other compounds such as (+)-catechin (in all cultivars) or quercetin-3-*O*-glucuronide (in Tannat variety) followed the inverse order. Skin extracts of all varieties at BV stage, have proven to be effective to decrease (>50%) NO and ROS intracellular production in macrophages. In all cases, our observations can only partially explain the observed effects. It is important to keep in mind that this work is based only on the polyphenolic composition, however grape skins represent a very complex matrix. Indeed, other components or nutrients such as vitamins can contributed substantially to the biological activities. In addition, we cannot forget the possible synergistic interactions between individual polyphenolic compounds or other bioactives present in grape skins. To conclude, more studies should be conducted in order to demonstrate the specific contribution of polyphenolics or other compounds to the antioxidant and anti-inflammatory activity of grape skins.

## Figures and Tables

**Table 1:** °Brix and pH of Merlot, Tannat and Syrah berries at before veraison (BV), veraison (V) and maturity (M) stages.

Grapes.	Merlot		Tannat		Syrah	
	°Brix	pH	°Brix	pH	°Brix	pH
BV	4.37	2.56	4.3	2.57	4.37	2.58
V	8.57	3.34	11.64	2.75	16.97	3.42
M	25.97	3.78	25.84	3.87	25.37	4

**Table 2:** Total polyphenol content (TPC) and antioxidant activity (measured by DPPH test) of Merlot, Tannat and Syrah skin samples at BV, V and M.

**TPC (** g GAE/kg of Skins FW) *			
**Skins**	**Merlot**	**Tannat**	**Syrah**
BV	18.5 ± 2.6 ^a^	19.2 ± 7.9 ^a^	18.3 ± 1.2 ^a^
V	17.0 ± 2.1 ^b^	16.7 ± 2.9 ^b^	16.9 ± 2.5 ^b^
M	13.1 ± 2.6 ^c^	16.5 ± 1.7 ^b^	16.2 ± 0.8 ^b^
**DPPH (g TE/k** g of skins FW) **			
BV	4.6 ± 0.4 ^b^	6.0 ± 0.6 ^b^	6.5 ± 0.4 ^b^
V	3.8 ± 1.3 ^c^	4.1 ± 0.9 ^ab^	5.9 ± 1.1 ^b^
M	3.3 ± 0.1 ^a^	3.9 ± 0.9 ^a^	2.9 ± 0.4 ^a^

* TPC expressed as values means ± SE (*n* = 9) of 3 biological replicates × 3 technical replicates. Different letters indicate the significant differences between stages according to Tukey’s test, *p* ˂ 0.05. ** Antioxidant activity expressed as µmol TE/g of skins (DW). Values means ± SE (*n* = 9) of 3 biological replicates × 3 technical replicates. Different letters indicate the significant differences between stages of ripening according to Tukey’s test, *p* ˂ 0.05.

**Table 3:** Quantification of individual flavan-3-ols, procyanidins, flavonols and stilbenes in skins extracts of Merlot, Tannat and Syrah varieties at different stages of ripening.

	Merlot			Tannat			Syrah		
	BV	V	M	BV	V	M	BV	V	M
Flavan-3-ols/procyanidins *									
(+)-Catechin	107.04 ± 1.48	53.36 ± 0.51	23.95 ± 0.95	114.45 ± 4.66	90.11 ± 5.18	29.43 ± 6.56	97.72 ± 16.66	111.11 ± 0.02	39.82 ± 5.84
(−)-Epicatechin	1.38 ± 0.44	5.30 ± 0.37	5.90 ± 2.47	2.32 ± 0.78	9.85 ± 0.96	7.31 ± 1.50	0.86 ± 0.08	30.94 ± 5.54	8.75 ± 0.30
Procyanidin B1	75.53 ± 3.90	128.23 ± 3.56	71.43 ± 2.14	50.77 ± 0.39	108.18 ± 5.16	105.36 ± 6.09	52.66 ± 4.24	138.63 ± 8.90	89.92 ± 9.03
Procyanidin B2	2.09 ± 0.36	3.83 ± 0.50	9.21 ± 0.76	3.07 ± 0.11	4.27 ± 0.03	4.50 ± 0.17	1.16 ± 0.10	6.82 ± 2.44	9.57 ± 0.20
Procyanidin B3	29.45 ± 2.48	22.99 ± 3.85	12.04 ± 1.74	17.35 ± 0.04	24.27 ± 2.34	6.99 ± 1.71	26.14 ± 2.95	30.26 ± 0.64	16.51 ± 3.56
Procyanidin B4	5.61 ± 0.33	5.14 ± 0.62	7.42 ± 2.07	7.35 ± 0.57	8.22 ± 0.87	4.16 ± 1.04	6.42 ± 1.17	7.78 ± 1.92	6.73 ± 1.20
Flavonols *									
Quercetin	0.13 ± 0.04	0.33 ± 0.06	0.28 ± 0.16	0.08 ± 0.04	0.25 ± 0.06	0.34 ± 0.09	0.07 ± 0.00	2.02 ± 0.30	1.03 ± 0.45
Quercetin-3-*O*-hexoside	2.78 ± 0.49	28.35 ± 5.83	38.07 ± 3.01	3.21 ± 0.65	6.09 ± 2.28	8.56 ± 0.11	2.61 ± 1.03	75.88 ± 1.34	148.13 ± 25.29
Quercetin-3-*O*-galactoside	0.62 ± 0.08	4.51 ± 1.06	3.99 ± 0.92	1.04 ± 0.10	0.87 ± 0.13	0.75 ± 0.05	0.30 ± 0.04	7.26 ± 0.66	9.66 ± 1.79
Quercetin-3-*O*-glucuronide	75.14 ± 9.01	154.63 ± 15.09	122.17 ± 6.80	98.93 ± 13.71	44.78 ± 4.22	58.10 ± 21.21	63.90 ± 0.75	227.90 ± 18.01	117.70 ± 12.62
Quercetin-3-*O*-rhamnoside	0.03 ± 0.02	1.06 ± 0.43	0.45 ± 0.18	0.01 ± 0.00	0.02 ± 0.00	0.02 ± 0.00	0.02 ± 0.00	1.16 ± 0.12	1.10 ± 0.84
Quercetin-3-*O*-rutinoside	1.33 ± 0.23	1.75 ± 0.11	0.49 ± 0.05	5.46 ± 0.78	3.34 ± 0.28	0.80 ± 0.46	0.38 ± 0.21	0.58 ± 0.50	1.22 ± 1.10
Kaempferol-3-*O*-glucoside	0.29 ± 0.10	6.78 ± 1.75	4.28 ± 0.10	0.40 ± 0.15	1.67 ± 0.52	0.32 ± 0.12	0.08 ± 0.05	3.87 ± 1.38	9.06 ± 2.37
Stilbenes *									
*Cis*-resveratrol	0.03 ± 0.02	0.04 ± 0.01	0.14 ± 0.02	0.03 ± 0.01	0.45 ± 0.26	0.09 ± 0.02	0.03 ± 0.00	1.94 ± 0.05	1.43 ± 0.65
*Trans*-resveratrol	0.11 ± 0.04	0.13 ± 0.02	0.25 ± 0.11	0.04 ± 0.01	0.44 ± 0.38	0.17 ± 0.10	0.13 ± 0.02	4.07 ± 1.21	5.34 ± 0.18
*∑ resveratrol*	0.15	0.17	0.39	0.07	0.89	0.26	0.16	6.01	6.77
*Cis*-piceid	0.30 ± 0.06	1.39 ± 0.44	9.63 ± 0.93	1.04 ± 0.48	2.36 ± 1.31	3.00 ± 1.12	0.73 ± 0.21	15.74 ± 6.84	41.99 ± 1.41
*Trans*-piceid	0.10 ± 0.02	0.36 ± 0.01	1.93 ± 0.37	0.68 ± 0.32	0.52 ± 0.25	1.41 ± 0.28	0.20 ± 0.08	2.12 ± 1.14	7.65 ± 1.53
*∑ piceid*	0.39	1.75	11.56	1.72	2.88	4.41	0.93	17.86	49.64
*Cis*-piceatannol	0.03 ± 0.01	0.38 ± 0.19	0.26 ± 0.07	0.05 ± 0.02	0.03 ± 0.00	0.09 ± 0.03	0.03 ± 0.00	9.18 ± 1.52	1.52 ± 0.53
*Trans*-piceatannol	0.02 ± 0.01	0.23 ± 0.11	1.38 ± 0.80	0.02 ± 0.01	0.02 ± 0.01	0.32 ± 0.15	0.02 ± 0.00	5.48 ± 0.91	10.49 ± 1.93
*∑ piceatannol*	0.04	0.61	1.64	0.06	0.23	0.41	0.05	14.66	12.01
*Cis*-astringin	0.03 ± 0.00	0.24 ± 0.00	2.93 ± 0.49	0.04 ± 0.02	0.13 ± 0.08	0.28 ± 0.01	0.04 ± 0.02	0.83 ± 0.33	3.77 ± 0.98
*Trans*-astringin	0.04 ± 0.01	0.72 ± 0.10	5.91 ± 1.14	0.09 ± 0.06	0.29 ± 0.16	1.70 ± 0.81	0.04 ± 0.02	1.97 ± 0.33	13.27 ± 1.03
*∑ astringin*	0.07	0.96	8.84	0.13	0.42	1.98	0.08	2.79	17.05
ε-viniferin	0.73 ± 0.19	0.93 ± 0.37	1.61 ± 0.26	0.12 ± 0.04	1.70 ± 0.70	0.63 ± 0.16	0.27 ± 0.11	1.38 ± 0.37	4.77 ± 0.21
ω-viniferin	0.03 ± 0.01	0.04 ± 0.01	0.10 ± 0.04	0.01 ± 0.000	0.07 ± 0.03	0.06 ± 0.01	0.01 ± 0.00	0.27 ± 0.05	0.95 ± 0.04
σ-viniferin	0.01 ± 0.00	0.01 ± 0.00	0.95 ± 0.14	0.01 ± 0.01	0.07 ± 0.02	0.08 ± 0.02	0.01 ± 0.00	0.84 ± 0.13	2.97 ± 0.70
Pallidol	0.02 ± 0.01	0.03 ± 0.01	1.88 ± 1.11	0.03 ± 0.01	0.48 ± 0.30	0.28 ± 0.05	0.05 ± 0.02	1.06 ± 0.46	6.73 ± 2.57
Parthenocissin A	0.06 ± 0.04	0.08 ± 0.07	2.37 ± 1.11	0.06 ± 0.04	0.53 ± 0.37	0.39 ± 0.14	0.05 ± 0.02	1.47 ± 0.18	4.81 ± 0.91
Miyabenol C	0.06 ± 0.01	0.01 ± 0.00	0.22 ± 0.02	0.08 ± 0.06	0.27 ± 0.15	0.16 ± 0.07	0.34 ± 0.02	0.57 ± 0.09	3.05 ± 0.85
Hopeaphenol	0.03 ± 0.01	0.06 ± 0.01	0.26 ± 0.04	0.01 ± 0.00	0.02 ± 0.00	0.03 ± 0.01	0.02 ± 0.00	0.05 ± 0.01	0.22 ± 0.08
Isohopeaphenol	0.03 ± 0.00	0.04 ± 0.01	0.44 ± 0.19	0.04 ± 0.01	0.04 ± 0.00	0.61 ± 0.07	0.03 ± 0.01	1.07 ± 0.31	2.95 ± 0.22

* Flavan-3-ols, procyanidins, flavonols and stilbenes are expressed as mg of their corresponding standard per kg of skins FW. Values means ± SE of 3 biological replicates.

**Table 4:** Quantification of individual anthocyanins in skins extracts of Merlot, Tannat, and Syrah varieties at different stages of ripening.

	Merlot			Tannat			Syrah		
	BV	V	M	BV	V	M	BV	V	M
**Anthocyanins**									
Delphinidin-3-*O*-glucoside	nd	269.52 ± 18.26	352.42 ± 27.39	nd	145.86 ± 72.87	395.07 ± 134.98	nd	526.08 ± 204.63	396.80 ± 151.81
Cyanidin-3-*O*-glucoside	nd	265.65 ± 64.55	346.87 ± 26.86	nd	100.56 ± 54.84	156.56 ± 41.43	nd	195.24 ± 105.11	143.69 ± 23.56
Petunidin-3-*O*-glucoside	nd	86.89 ± 69.48	348.83 ± 17.31	nd	218.86 ± 11.05	624.99 ± 136.52	nd	1090.05 ± 660.16	543.70 ± 184.11
Peonidin-3-*O*-glucoside	nd	801.77 ± 173.48	1185.78 ± 94.42	nd	176.48 ± 117.35	324.17 ± 33.72	nd	742.67 ± 312.21	709.13 ± 109.47
Malvidin-3-*O*-glucoside	nd	993.74 ± 238.54	1378.45 ± 69.64	nd	635.40 ± 389.99	1948.62 ± 204.63	nd	2362.31 ± 339.03	2321.65 ± 682.10
***∑ 3-O-glucosides***	-	2617.58	3612.86	-	1277.18	3449.42	-	4916.36	4114.97
Petunidin-3-*O*-(6-*O*-acetyl)-glucoside	nd	107.48 + 19.38	125.87 + 3.82	nd	58.61 + 24.12	160.74 + 42.68	nd	185.95 + 37.20	172.08 + 34.93
Peonidin-3-*O*-(6-*O*-acetyl)-glucoside	nd	200.81 ± 32.21	300.34 ± 36.02	nd	57.46 ± 20.84	94.67 ± 12.26	nd	279.77 ± 60.58	287.06 ± 18.66
Malvidin-3-*O*-(6-*O*-acetyl)-glucoside	nd	385.37 ± 78.51	572.86 ± 14.73	nd	123.42 ± 67.78	427.06 ± 60.19	nd	785.32 ± 70.90	978.46 ± 203.50
***∑ 3-O-acetylglucosides***	-	693.66	999.07	-	239.49	682.46	-	1251.04	1437.6
Delphinidin-3-*O*-(6-*O*-*p*-coumaroyl)-glucoside	nd	83.91 + 15.15	134.52 + 8.02	nd	53.52 + 20.15	92.14 + 9.06	nd	151.28 + 22.97	164.11 + 8.47
Cyanidin-3-*O*-(6-*O*-*p*-coumaroyl)-glucoside	nd	105.93 + 15.5	126 + 7.63	nd	66.31 + 26.66	160.24 + 24.22	nd	237.91 + 16.67	226.65 + 66.26
Peonidin-3-*O*-(6-*O*-*p*-coumaroyl)-glucoside	nd	153.37 ± 22.60	250.27 ± 31.35	nd	59.60 ± 24.76	118.29 ± 10.51	nd	444.01 ± 83.32	451.89 ± 70.38
Malvidin-3-*O*-(6-*O*-*p*-coumaroyl)-glucoside	nd	342.07 ± 59.97	585.13 ± 48.62	nd	143.01 ± 80.71	470.23 ± 63.55	nd	905.34 ± 126.90	1183.21 ± 385.23
***∑ 3-O-coumaroylglucosides***	-	685.29	1095.91	-	322.44	840.9	-	1738.19	2025.86

**Table 5:** Mean degree of polymerization (mDP) values of skins extracts of Merlot, Tannat and Syrah varieties at different stages of ripening.

mDP			
Skins	Merlot	Tannat	Syrah
BV	18.05 ± 0.07	13.88 ± 0.48	18.11 ± 0.40
V	32.21 ± 0.94	15.52 ± 0.01	17.62 ± 0.07
M	25.00 ± 0.13	16.08 ± 0.44	25.82 ± 0.90

mDP expressed as values means ± SE (*n* = 9) of 3 biological replicates × 3 technical replicates.

## Supplementary Materials

The following are available online at https://www.mdpi.com/2304-8158/10/3/541/s1, Figure S1: MRM chromatograms of identified and quantified compounds. Figure S2: LC-DAD chromatogram of anthocyanins identified and quantified in samples.
