# Supplementary material for: Polyphenolic Characterization of Merlot, Tannat and Syrah Skin Extracts at Different Degrees of Maturity and Anti-Inflammatory Potential in RAW 264.7 Cells
Source: Foods. 2021 Mar 5;10(3):541. doi: 10.3390/foods10030541 (PMC7998996; doi:10.3390/foods10030541)
Supplement: Supplementary file 1 [file foods-10-00541-s001.pdf]

Supplementary materials

Figure S1. MRM chromatograms of identified and quantified compounds.

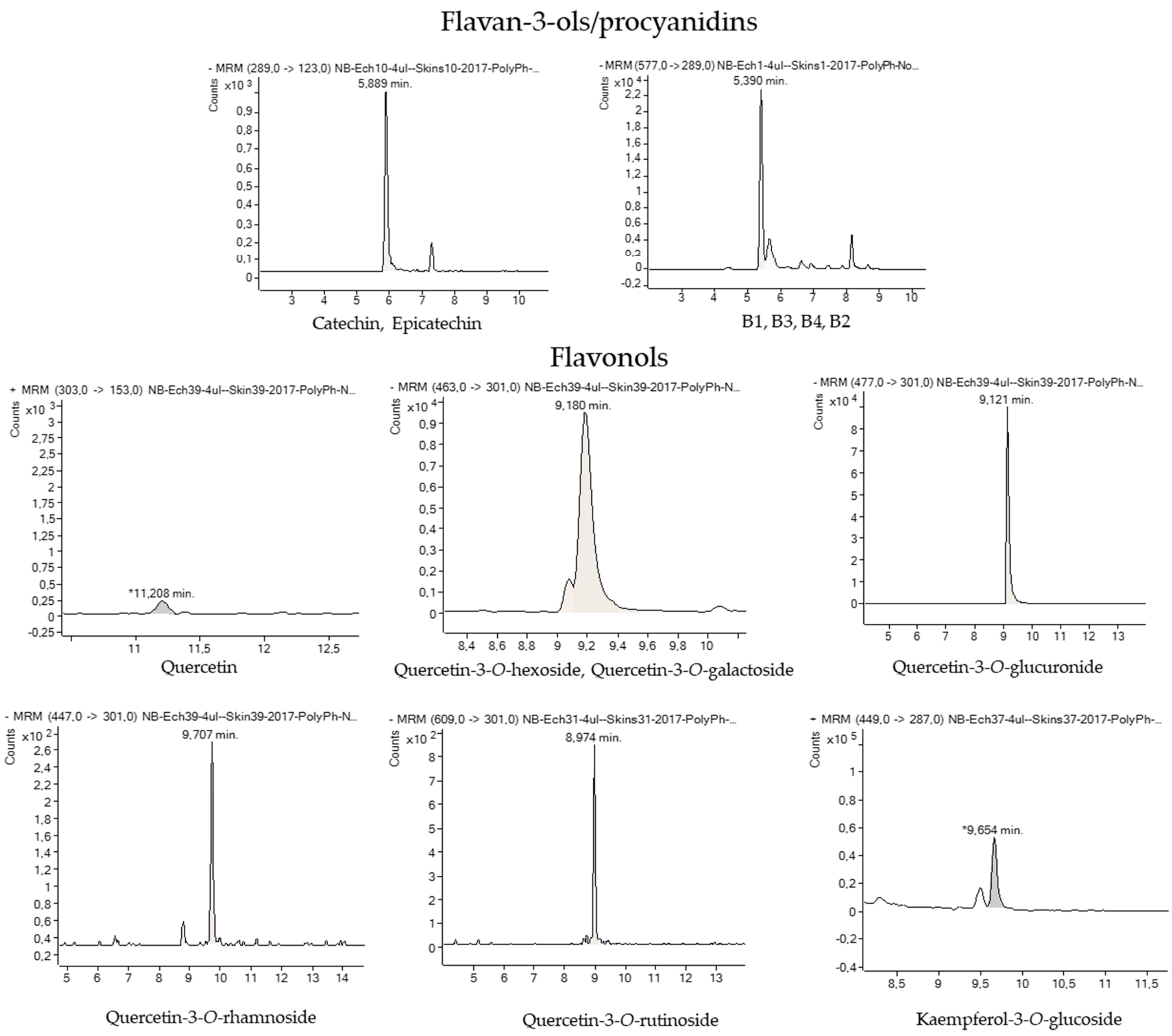

Stilbenes

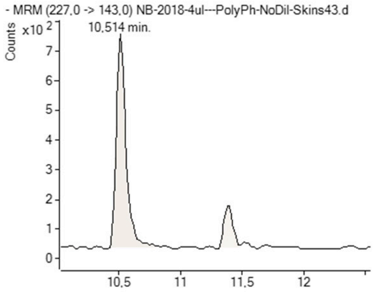

trans-resveratrol, *cis*-resveratrol

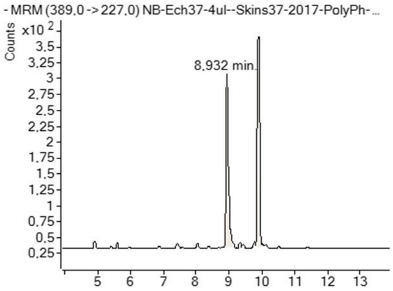

trans-piceid, *cis*-piceid

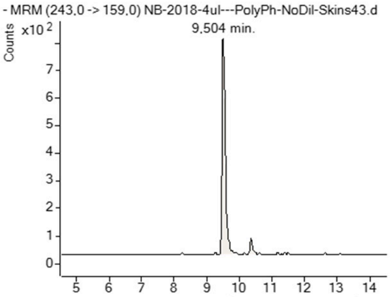

trans-piceatannol, *cis*-piceatannol

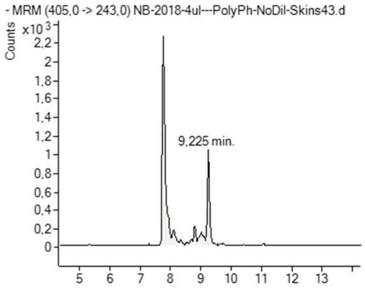

trans-astringin, *cis*-astringin

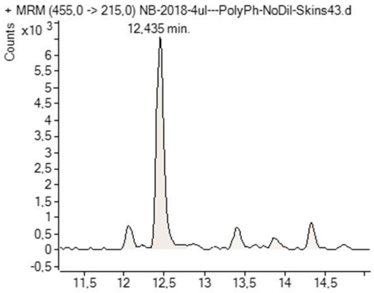

ε-viniferin, ω-viniferin, σ-viniferin

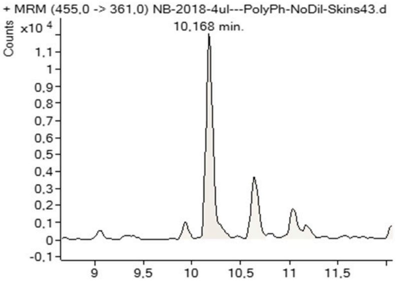

Pallidol, Parthenocissin

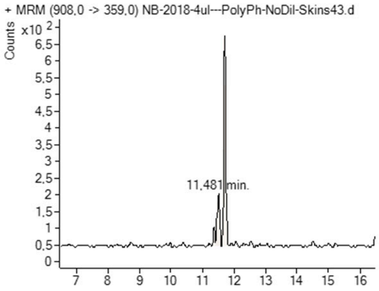

Hopeaphenol, Isohopeaphenol

Figure S2. LC-DAD chromatogram of anthocyanins identified and quantified in samples.

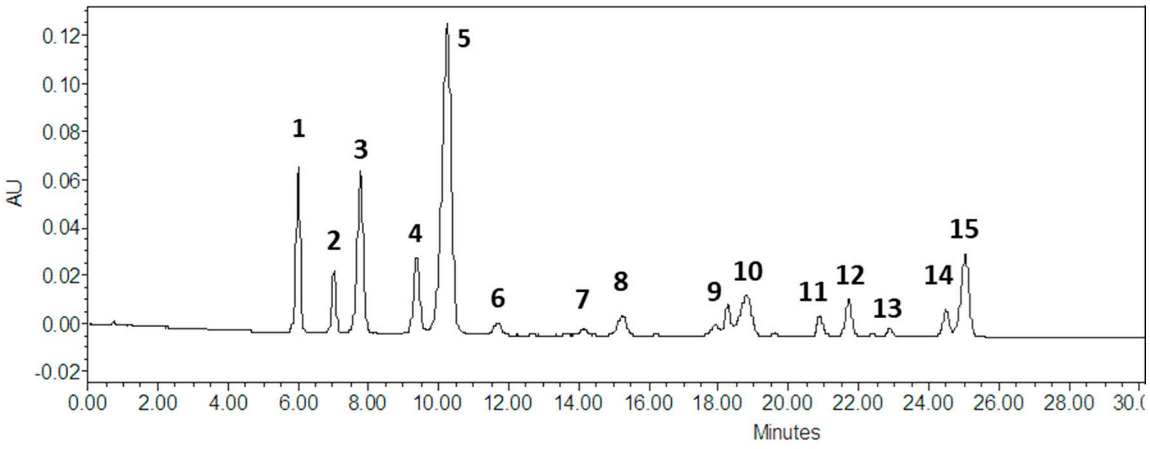

1: Delphinidin-3-*O*-glucoside; **2:** Cyanidin-3-*O*-glucoside; **3:** Petunidin-3-*O*-glucoside; **4:** Peonidin-3-*O*-glucoside; **5:** Malvidin-3-*O*-glucoside; **6:** Delphinidin-3-*O*-(6-*O*-acetyl)-glucoside; **7:** Cyanidin-3-*O*-(6-*O*-acetyl)-glucoside; **8:** Petunidin-3-*O*-(6-*O*-acetyl)-glucoside; **9:** Peonidin-3-*O*-(6-*O*-acetyl)-glucoside; **10:** Malvidin-3-*O*-(6-*O*-acetyl)-glucoside; **11:** Delphinidin-3-*O*-(6-*O*-*p*-coumaroyl)-glucoside; **12:** Cyanidin-3-*O*-(6-*O*-*p*-coumaroyl)-glucoside; **13:** Petunidin-3-*O*-(6-*O*-*p*-coumaroyl)-glucoside; **14:** Peonidin-3-*O*-(6-*O*-*p*-coumaroyl)-glucoside; **15:** Malvidin-3-*O*-(6-*O*-*p*-coumaroyl)-glucoside.
